# Patient Survival Between Hemodialysis and Peritoneal Dialysis Among End-Stage Renal Disease Patients Secondary to Myeloperoxidase-ANCA-Associated Vasculitis

**DOI:** 10.3389/fmed.2021.775586

**Published:** 2022-01-14

**Authors:** Xueqin Wu, Yong Zhong, Ting Meng, Joshua Daniel Ooi, Peter J. Eggenhuizen, Rong Tang, Wannian Nie, Xiangcheng Xiao, Jian Sun, Xiang Ao, Hao Zhang

**Affiliations:** ^1^Department of Nephrology, The Third Xiangya Hospital, Central South University, Changsha, China; ^2^Department of Nephrology, Xiangya Hospital, Central South University, Changsha, China; ^3^Centre for Inflammatory Diseases, Monash University, Clayton, VIC, Australia

**Keywords:** AAV (ANCA-associated vasculitis), ESRD (end-stage renal disease), hemodialysis (HD), peritoneal dialysis (PD), patient survival

## Abstract

**Background:**

A significant proportion of anti-neutrophil cytoplasmic antibody (ANCA) associated glomerulonephritis eventually progresses to end-stage renal disease (ESRD) thus requiring long-term dialysis. There is no consensus about which dialysis modality is more recommended for those patients with associated vasculitis (AAV-ESRD). The primary objective of this study was to compare patient survival in patients with AAV-ESRD treated with hemodialysis (HD) or peritoneal dialysis (PD).

**Methods:**

This double-center retrospective cohort study included dialysis-dependent patients who were treated with HD or PD. Clinical data were collected under standard format. The Birmingham vasculitis activity score (BVAS) was used to evaluate disease activity at diagnosis and organ damage was assessed using the vasculitis damage index (VDI) at dialysis initiation.

**Results:**

In total, 85 patients were included: 64 with hemodialysis and 21 with peritoneal dialysis. The patients with AAV-PD were much younger than the AAV-HD patients (48 vs. 62, *P* < 0.01) and more were female (76.2 vs. 51.6%, *P* = 0.05). The laboratory data were almost similar. The comorbidities, VDI score, and immuno-suppressive therapy at dialysis initiation were almost no statistical difference. Patient survival rates between HD and PD at 1 year were 65.3 vs. 90% (*P* = 0.062), 3 year were 59.6 vs. 90% (*P* < 0.001), and 5 years were 59.6 vs. 67.5% (*P* = 0.569). The overall survival was no significant difference between the two groups (*P* = 0.086) and the dialysis modality (HD or PD) was not shown to be an independent predictor for all-cause death (hazard ratio (HR) 0.73; 95% confidence interval (CI) 0.31–1.7; *P* = 0.473). Cardio-cerebrovascular events were the main cause of death among AAV-HD patients while infection in patients with AAV-PD.

**Conclusion:**

These results provide real-world data that the use of either hemodialysis or peritoneal dialysis modality does not affect patient survival for patients with AAV-ESRD who need long-term dialysis.

## Introduction

Anti-neutrophil cytoplasmic antibody-associated glomerulonephritis (ANCA-GN) is one of the major complications of anti-neutrophil cytoplasmic antibody (ANCA)-associated vasculitis (AAV). The myeloperoxidase (MPO)-ANCA AAV which is the most common subtype among Chinese AAV patients is more likely to get kidney involvement than proteinase 3 (PR3)-ANCA AAV (1). The renal outcomes in patients with AAV have improved significantly over the past decades, however, a significant proportion (up to 20–30%) of them eventually progress to end-stage renal disease (ESRD) requiring dialysis, especially in patients without timely and adequate immunosuppressive therapy at the initial presentation (2, 3).

Renal replacement therapy (RRT) including hemodialysis (HD), peritoneal dialysis (PD), and kidney transplantation (KT) is the established treatment for those patients with AAV-ESRD and it seems that they were more likely (4-fold) to receive HD than PD (4). Previous studies have mainly focused on the renal survival or patient survival of AAV before developing ESRD (5–7). Few studies reported the condition of AAV patients needing long-term dialysis after reaching ESRD. In a large cohort study in France, the survival of AAV patients in chronic dialysis did not differ significantly from non-vasculitis patients on dialysis (8). At the same time, researchers of Spain compared the outcomes of different RRT on pauci-immune vasculitis and also reported that dialysis outcome seems equal to other causes of chronic kidney disease (9). Yet, due to the limited sample size, they could not draw a solid conclusion of which RRT modality is better for patients with AAV-ESRD. Up to now, there is no consensus or guideline about which dialysis modality is more suitable for those patients with AAV-ESRD (10).

Thus, we conducted this retrospective cohort study with a larger sample size aiming to compare the difference in patient survival and evaluate the cause of death between HD and PD in a population of ESRD patients secondary to MPO-AAV.

## Methods

### Patients

The Hunan Vasculitis Study Group conducted this retrospective study and recruited patients who were diagnosed with AAV-ESRD and needed long-term dialysis from January 2010 to December 2020 in the Department of Nephrology, Xiangya Hospital, and the Third Xiangya Hospital. All patients fulfilled the 2012 Chapel Hill Consensus Conferences Nomenclature of vasculitis and were then classified following the algorithm suggested by the European Medicines Agency in 2007. Exclusion criteria were as follows: (1) eosinophilic granulomatosis with polyangiitis (EGPA) or secondary vasculitis; (2) comorbid kidney diseases, such as IgA nephropathy, membranous nephropathy, diabetic nephropathy, and anti-glomerular basement membrane glomerulonephritis; (3) the coexistence of another autoimmune disease, such as lupus nephritis. The study protocol followed the provisions of the Declaration of Helsinki and was approved by the ethics committees of both hospitals.

### Data Collection

Patients' clinical data were derived from the electronic medical system and the sharing online PD Big Data Remote Management Platform of Hunan province. All electronic data were collected by one researcher under a standard format and the follow-up data were fulfilled by connecting the patients or relatives as they may be censored from our medical system or platform. The demographic data and laboratory parameters were picked and analyzed using the most recent serological results before dialysis initiation. The estimated glomerular filtration rate (eGFR) was calculated as described previously (11). The Birmingham vasculitis activity score (BVAS, range 0–63, v. 3) was calculated at the time of AAV diagnosis while the vasculitis damage index (VDI, range 0–64) was assessed at the time of dialysis. The outcome was defined as the time elapsed between the dialysis initiation and all-cause death or the endpoint of observation. For patients who did not die during the observation period, switched to HD of PD patients, and received kidney transplantation were censored. Date and cause of death when available were recorded.

### Statistical Analysis

Continuous variables are presented as mean ± *SD* or median (interquartile range, IQR) and categorical variables are presented as counts and percentages. Student's *t*-test for normally distributed variables or the Mann–Whitney U test for non-normally distributed data was used to compare groups. Chi-square test and Fisher exact test were used for categorical variables. Patient survival was studied with Kaplan–Meier method and assessed with univariate and multivariate logistic regression analysis. Statistical significance was defined as *P* < 0.1. Analyses were performed using the SPSS statistical software (version 23.085, SPSS Inc., Chicago, Ill., USA) and R_4.1.2 (https://www.r-project.org/).

## Results

### Baseline Characteristics of Patients With AAV-ESRD

This study enrolled 85 patients with MPO-AAV and undergoing long-term dialysis shown in Table 1. The ratio of patient number was almost 3:1 (HD, 64 vs. PD, 21). Both groups were predominantly female with 51.6% in the HD and 76.2% in the PD groups (*P* = 0.05). Age at dialysis onset between groups was 62 ± 12.2 and 48.4 ± 17.4 (*P* < 0.01). Only Platelet was found to be significant (*P* = 0.02) while other observed serological variables were similar between groups.

**Table 1:** Baseline characteristics of patients with AAV-ESRD at start of dialysis.

** Variables**	**All patients (*n =* 85)**	**Haemodialysis (*n =* 64, 75%)**	**Peritoneal dialysis (*n =* 21, 25%)**	***P-*value**
Age, mean ± SD, years	59 ± 15	62 ± 12	48 ± 17	<0.01
Male (%)	36 (42.6%)	31 (48.4%)	5 (23.8%)	0.05
**Laboratory data, mean** **±SD**				
Serum-creatinine, μmol/L	756.6 ± 248.2	758.2 ± 257.2	752.1 ± 224.4	0.92
Urea-nitrogen, mmol/L	26.0 ± 9.8	26.0 ± 10.4	25.9 ± 7.5	0.99
eGFR, ml/min/1.73 m^2^	6.0 ± 2.3	6.0 ± 2.1	6.1 ± 2.7	0.80
White blood cells, 10^9^/L	8.1 ± 3.3	8.2 ± 3.5	7.9 ± 2.6	0.71
Hemoglobin, g/L	74 ± 16.1	74.2 ± 15.6	73.4 ± 18.0	0.84
Platelet, 10^9^ /L	201.9 ± 86.2	211.5 ± 92.8	173.0 ± 54.1	0.02
ESR, mm/h	68.9 ± 38.8	70.9 ± 39.6	61.2 ± 35.5	0.38
CRP, mg/dl	9.5 ± 5.8	8.9 ± 5.1	9.7 ± 6.0	0.60
**Comorbidities**, ***n*** **(%)**				
CVD	24 (28.2%)	13 (20.3%)	11 (52.4%)	<0.01
Pulmonary disease	12 (14.1%)	12 (18.8%)	4 (19.0%)	0.98
Diabetes mellitus	6 (7.1%)	5 (7.8%)	1 (4.8%)	0.64
Hepatitis	3 (3.5%)	1 (1.6%)	2 (9.5%)	0.09
VDI score, mean ± SD	1.7 ± 0.7	1.7 ± 0.7	1.8 ± 0.7	0.54
**Immunosuppressive therapy**, ***n*** **(%)**				
Corticosteroids <7.5 mg/d	67 (78.8%)	50 (78.1%)	17 (81%)	0.78
Corticosteroids ≥7.5 mg/d	6 (7.1%)	5 (7.8%)	1 (4.8%)	0.64
Corticosteroids +CYC	4 (4.7%)	3 (4.7%)	1 (4.8%)	0.99
Other	8 (9.4%)	6 (9.4%)	2 (9.5%)	0.98

*ESR, erythrocyte sedimentation rate; CRP, C-reactive protein; CVD, cardiovascular disease. VDI, Vasculitis Damage Index (range 1–3); CYC, cyclophosphamide*.

As for comorbidities at dialysis initiation, no statistically significant differences were found for diabetes mellitus, pulmonary disease, and hepatitis. cardio- vascular disease including hypertension, coronary heart disease, and heart failure was different between groups (*P* < 0.01).

After induction therapy, most patients with AAV-ESRD were achieved extrarenal remission. Details of extrarenal organ involvement and BVAS at diagnosis of AAV were presented in Supplementary Table S1. Corticosteroids (< 7.5 mg/d) with mycophenolate, tripterygium glycosides, or azathioprine were the dominant maintenance therapy in our cohort (78.8%). Still, about 11.8 percentage of patients undertook high doses of corticosteroids with or without cyclophosphamide suffered from Goodpasture's disease or had extrarenal organ involvement. The VDI score at dialysis onset ranged from 1 to 3 and no statistically significant differences were found between groups.

### Overall Survival on Dialysis

The median follow-up of the HD group was 19 months while 30 months of the PD group. During the follow-up, five PD patients switched to hemodialysis and one patient received kidney transplantation and the graft still functions well at the end of observation. No kidney transplantations were performed in the HD group and no HD patient changed to peritoneal dialysis.

**Figure 1:**
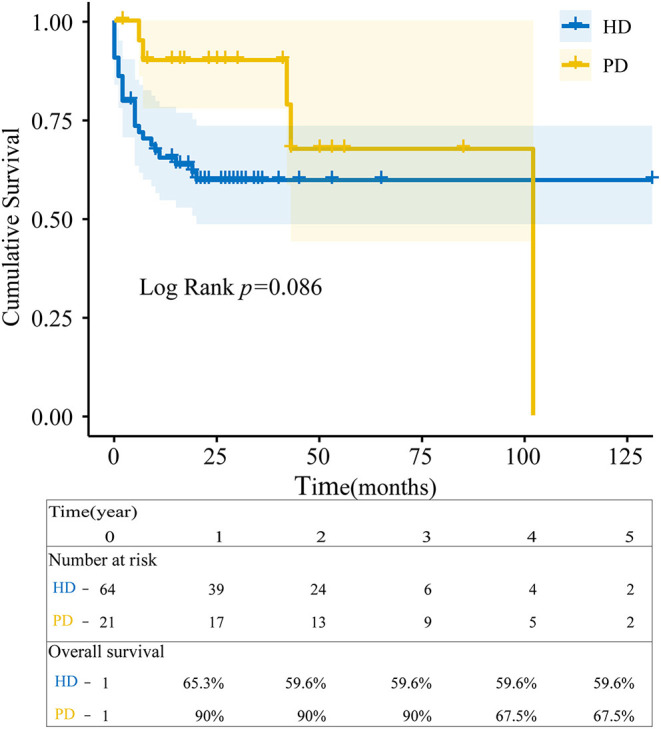
Overall survival estimates since dialysis initiation. The blue line represented patients with ANCA-associated vasculitis end-stage renal disease (AAV-ESRD) receiving hemodialysis (HD) and the yellow line with peritoneal dialysis (PD). Survival between HD and PD patients was analyzed by the Kaplan-Meier method using R language (*p* = 0.086).

Survival rates of AAV-HD at 1, 3, and 5 years were 65.3, 59.6, and 59.6% and 90, 90, and 67.5% of AAV-PD. Almost 50% of AAV-HD patients died in the first three years. The overall survival between the two groups was no significant difference (*P* = 0.086) at the end of observation (Figure 1). Thirty deaths were recorded. Infection, cardio-cerebrovascular events, and carcinoma were the main causes of death in patients with AAV-ESRD depending on long-term dialysis as shown in Table 2.

**Table 2:** Causes of death in patients with AAV-ESRD.

	**All patients,** ***n =* 30**	**Haemodialysis,** ***n =* 25**	**Peritoneal dialysis,** ***n =* 5**
Infection	9 (28%)	5 (20%)	4 (80%)
Heart failure	10 (31%)	9 (36%)	1 (20%)
Gastrointestinal hemorrhage	3 (9%)	3 (12%)	0
hemorrhagic stroke	4 (13%)	4 (16%)	0
Hyperkalemia	2 (6%)	2 (8%)	0
Carcinoma	2 (6%)	2 (8%)	0

*Results are expressed as n (%)*.

### Predictors Associated With Mortality

**Figure 2:**
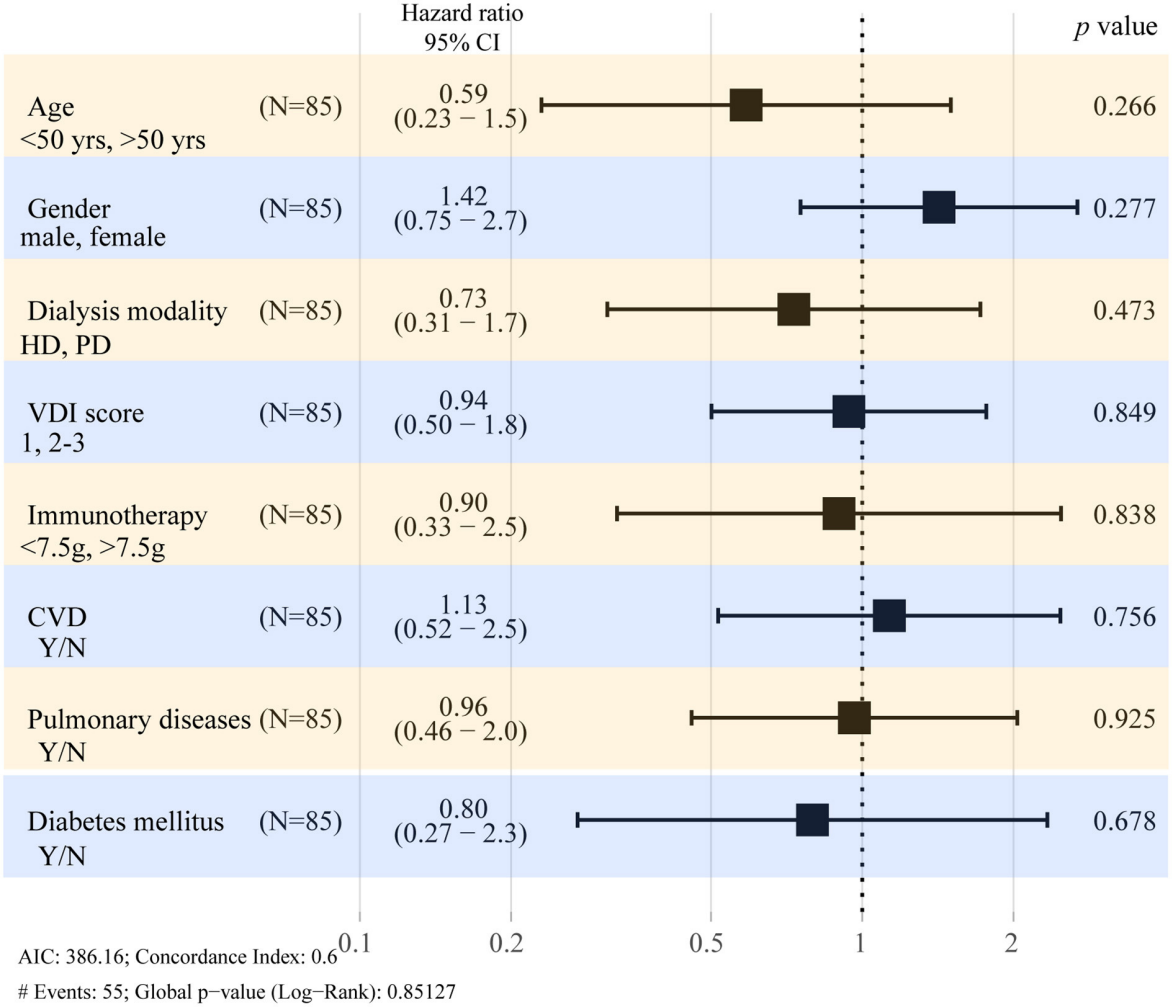
Hazard ratios for death in AAV-ESRD patient with long-term dialysis. Forest plots show the results of multivariate analysis of predictors at dialysis initiation. CI, confidence interval; HD, hemodialysis; PD, peritoneal dialysis; VDI, Vasculitis Damage Index; CVD, cardiovascular disease.

In order to assess factors that could affect the outcome of this study, variables considered clinically important were included in the multivariate model. As shown in Table 3 and Figure 2, there was no difference between HD or PD modality in terms of the survival of patients with AAV-ESRD when adjusted for age, sex, VDI score, comorbidities, and drug treatment at the start of dialysis.

**Table 3:** Predictors for death of AAV-ESRD patient with long-term dialysis.

** Variables**	**Univariate_cox**		**Multivariate_cox**	
	**HR (95% CI)**	***P-*value**	**HR (95% CI)**	***P-*value**
Age (ref. <50yrs)	2.6 (0.61_11)	0.2	0.59 (0.23–1.5)	0.27
Gender (ref. male)	0.89 (0.43_1.8)	0.75	1.4 (0.75–2.7)	0.28
Dialysis modality (ref. HD)	0.53 (0.2_1.4)	0.19	0.73 (0.31–1.7)	0.47
VDI score	0.77 (0.45_1.3)	0.32	0.94 (0.5–1.8)	0.85
Immunotherapy	1.5 (0.54_3.9)	0.46	0.9 (0.33–2.5)	0.84
CVD	0.89 (0.39_2)	0.78	1.1 (0.52–2.5)	0.76
Pulmonary diseases	0.74 (0.33_1.7)	0.48	0.96 (0.46–2)	0.93
Diabetes mellitus	0.25 (0.033_1.9)	0.18	0.8 (0.27–2.3)	0.68

*HD, hemodialysis; VDI, Vasculitis Damage Index; CVD, cardiovascular disease*.

## Discussion

Improvements in induction therapy for AAV have reduced the prevalence of AAV-related ESRD. However, there is little evidence to guide dialysis strategies for patients with AAV-ESRD currently. Therefore, we conducted this study for the sake of obtaining more clinical evidence to determine whether HD or PD is associated with better patient survival. Patients with AAV are more likely to receive HD compared to PD (12). In our cohort, more than 75% of patients with AAV-ESRD received HD which is similar to previous studies (9, 13). This selection was similar to the SLE-ESRD because immunosuppression could increase the morbidity of infection and result in technique failure of PD (14).

The overall survival rate was similar between patients with AAV-HD and with AAV-PD, which was similar to Chen YX's study and the comparative study of patients with SLE-ESRD initiating with PD vs. HD conducted by Contreras G, et al. (14). However, the survival rates of patients with AAV-PD were 90% at 1 year and 68.6% at 5 years, which was higher than survival rates observed by Chen YX, et al. The most plausible explanation is because most patients with AAV-PD in our cohort were much younger (48.4 ± 17.4 vs. 61.2 ± 12.4) as older age has been reported to be an adverse prognostic factor (15, 16).

Infection was the major cause of drop-out and mortality in patients with AAV-PD. Five patients with AAV-PD switched to HD due to refractory peritonitis. No patient with AAV-PD in our cohort experienced relapse during the PD process as one may argue that infection could increase the risk of relapse as SLE did (17, 18). The lower relapse rate in this study was partly due to the ANCA subtype (PR3-ANCA+ patients relapse more than MPO-ANCA+ patients) as our cohorts were MPO-ANCA+ patients and most patients with AAV-PD except one who received kidney transplantation ceased immunosuppression without active extrarenal manifestation of vasculitis after undergoing PD (19, 20).

The present study has several limitations. First, it is a retrospective analysis encompassing 10 years of clinical observation, treatment regimens at induction therapy were not standardized though there is no statistical significance regarding immunosuppressive therapy in this study. Moreover, with the implication of new regimens like rituximab, details of maintenance therapy, and disease flare-up were also needed to be discussed in further prospective cohort studies. Even so, to our knowledge, this study is so far the larger series of long-term follow-up in AAV-ESRD focused on different dialysis modalities.

In conclusion, this study demonstrates that HD and PD are two comparable dialysis modalities for patients with AAV-ESRD.

## Data Availability Statement

The original contributions presented in the study are included in the article Supplementary Material, further inquiries can be directed to the corresponding author/s.

## Ethics Statement

The studies involving human participants were reviewed and approved by the Ethics Committee of both hospitals. The patients/participants provided their written informed consent to participate in this study.

## Author Contributions

XW, YZ, XX, XA, and HZ designed the study. XW and RT collected and entered data. XW and TM analyzed the data. WN and JS contributed to the data acquisition and interpretation. XW and YZ wrote the original draft. JO, PE, XA, and HZ edited. All authors read and approved the final manuscript.

## Funding

This work was funded by the National Natural Science Foundation of China (No. 81870498 to HZ and No.81870499 to JS), the National Key R&D Program of China (2020YFC2005000 to XX), the Key Research and Development Program of Hunan province (2018WK2060 to XX and 2020WK2008 to YZ), the science and technology innovation Program of Hunan Province (2020RC5002 to JO), the Natural Science Foundation of Hunan Province (2019JJ40515 to WN), Chinese Society of Nephrology (18020010780 to YZ), the Fundamental Research Funds for the Central Universities of Central South University (2019zzts909 to XW).

## Conflict of Interest

The authors declare that the research was conducted in the absence of any commercial or financial relationships that could be construed as a potential conflict of interest.

## Publisher's Note

All claims expressed in this article are solely those of the authors and do not necessarily represent those of their affiliated organizations, or those of the publisher, the editors and the reviewers. Any product that may be evaluated in this article, or claim that may be made by its manufacturer, is not guaranteed or endorsed by the publisher.
