# Supplementary material for: Patient Survival Between Hemodialysis and Peritoneal Dialysis Among End-Stage Renal Disease Patients Secondary to Myeloperoxidase-ANCA-Associated Vasculitis
Source: Front Med (Lausanne). 2022 Jan 14;8:775586. doi: 10.3389/fmed.2021.775586 (PMC8804699; doi:10.3389/fmed.2021.775586)
Supplement: Supplementary file 1 [file Table_1.docx]

**Table S1: Extrarenal organ involvement at diagnosis of AAV**

| Variables | All patients  (n=85) | Haemodialysis（n=64, 75%） | Peritoneal dialysis  (n=21, 25%) | *P-value* |
| --- | --- | --- | --- | --- |
| General* | 28 (32.9%) | 20 (31.3%) | 8 (38.1%) | 0.56 |
| Mucous membranes,ENT | 5 (5.9%) | 2 (3.1%) | 3 (14.3%) | 0.06 |
| Nervous system | 5 (5.9%) | 4 (6.3%) | 1 (4.8%) | 0.80 |
| Chest | 11 (12.9%) | 8 (12.5%) | 3 (14.3%) | 0.83 |
| Cardiovascular | 4 (4.7%) | 2 (3.1%) | 2 (9.5%) | 0.23 |
| Abdominal | 8 (9.4%) | 6 (9.4%) | 2 (9.5%) | 0.98 |
| BVAS, median (IQR) | 18 (16-21) | 19 (17.8-23.0) | 18 (14-20) | 0.04 |

*Including [muscle](C:/Users/Administrator/AppData/Local/youdao/dict/Application/8.9.9.0/resultui/html/index.html#/javascript:;) [pain](C:/Users/Administrator/AppData/Local/youdao/dict/Application/8.9.9.0/resultui/html/index.html#/javascript:;), [arthrodynia](C:/Users/Administrator/AppData/Local/youdao/dict/Application/8.9.9.0/resultui/html/index.html#/javascript:;)/[arthritis](C:/Users/Administrator/AppData/Local/youdao/dict/Application/8.9.9.0/resultui/html/index.html#/javascript:;), fever（≥38 ℃）and weight loss(≥2 Kg); BVAS, Birmingham Vasculitis Activity Score (range 0–63); IQR, interquartile range.
